# A practice change intervention to improve antenatal care addressing alcohol consumption by women during pregnancy: research protocol for a randomised stepped-wedge cluster trial

**DOI:** 10.1186/s13012-018-0806-x

**Published:** 2018-08-20

**Authors:** Melanie Kingsland, Emma Doherty, Amy E. Anderson, Kristy Crooks, Belinda Tully, Danika Tremain, Tracey W. Tsang, John Attia, Luke Wolfenden, Adrian J. Dunlop, Nicole Bennett, Mandy Hunter, Sarah Ward, Penny Reeves, Ian Symonds, Chris Rissel, Carol Azzopardi, Andrew Searles, Karen Gillham, Elizabeth J. Elliott, John Wiggers

**Affiliations:** 1 Hunter New England Population Health, Hunter New England Local Health District, Wallsend, New South Wales Australia; 2 School of Medicine and Public Health, The University of Newcastle, Callaghan, New South Wales Australia; 3 Hunter Medical Research Institute, New Lambton Heights, New South Wales Australia; 4 Menzies School of Health Research, Charles Darwin University, Darwin, Northern Territory Australia; 5 School of Medicine, The University of Sydney, Camperdown, New South Wales Australia; 6 Sydney Children’s Hospital Network, Kids’ Research Institute, Westmead, New South Wales Australia; 7 Drug and Alcohol Clinical Services, Hunter New England Local Health District, Newcastle, New South Wales Australia; 8 Maternity and Gynaecology John Hunter Hospital, New Lambton Heights, New South Wales Australia; 9 Foundation for Alcohol Research and Education, Deakin, Australian Capital Territory Australia; 10 Adelaide Medical School, The University of Adelaide, Adelaide, South Australia Australia; 11 New South Wales Office of Preventive Health, Liverpool, New South Wales Australia

**Keywords:** Maternal, Alcohol consumption, Pregnancy, Antenatal care, Implementation, Clinical practice change, Stepped-wedge trial, Protocol

## Abstract

**Background:**

Despite clinical guideline recommendations, implementation of antenatal care addressing alcohol consumption by pregnant women is limited. Implementation strategies addressing barriers to such care may be effective in increasing care provision. The aim of this study is to examine the effectiveness, cost and cost-effectiveness of a multi-strategy practice change intervention in increasing antenatal care addressing the consumption of alcohol by pregnant women.

**Methods:**

The study will be a randomised, stepped-wedge controlled trial conducted in three sectors in a health district in New South Wales, Australia. Stepped implementation of a practice change intervention will be delivered to sectors in a random order to support the introduction of a model of care for addressing alcohol consumption by pregnant women. A staged process was undertaken to develop the implementation strategies, which comprise of: leadership support, local clinical practice guidelines, electronic prompts and reminders, opinion leaders, academic detailing (audit and feedback), educational meetings and educational materials, and performance monitoring. Repeated cross-sectional outcome data will be gathered weekly across all sectors for the study duration. The primary outcome measures are the proportion of antenatal appointments at ‘booking in’, 27–28 weeks gestation and 35–36 weeks gestation for which women report (1) being assessed for alcohol consumption, (2) being provided with brief advice related to alcohol consumption during pregnancy, (3) receiving relevant care for addressing alcohol consumption during pregnancy, and (4) being assessed for alcohol consumption and receiving relevant care. Data on resources expended during intervention development and implementation will be collected. The proportion of women who report consuming alcohol since knowing they were pregnant will be measured as a secondary outcome.

**Discussion:**

This will be the first randomised controlled trial to evaluate the effectiveness, cost and cost-effectiveness of implementation strategies in improving antenatal care that addresses alcohol consumption by pregnant women. If positive changes in clinical practice are found, this evidence will support health service adoption of implementation strategies to support improved antenatal care for this recognised risk to the health and wellbeing of the mother and child.

**Trial registrations:**

Australian and New Zealand Clinical Trials Registry, No. ACTRN12617000882325 (date registered: 16/06/2017).

## Background

Maternal alcohol consumption during pregnancy is associated with a number of adverse obstetric, fetal and child outcomes with lifelong consequences. These include, Fetal Alcohol Spectrum Disorder (FASD), miscarriage, stillbirth, preterm birth, congenital anomalies, and low birth weight [1]. No safe level of prenatal alcohol exposure has been established for the foetus and therefore many countries, including Australia, have national guidelines recommending that the safest option is for women to abstain from alcohol consumption when trying to conceive, during conception and during pregnancy [1–3].

Despite this recommendation, 10% of women worldwide consume alcohol during pregnancy, with notably higher prevalence estimates in regions with high levels of general alcohol consumption, such as the World Health Organisation European Region (prevalence estimate of alcohol consumption in pregnancy, 25%) [4]. In Australia, national surveys and prospective cohort studies report the prevalence of maternal alcohol consumption at any time during pregnancy to be between 35 and 72% [5–10]. For example, a prospective cohort study of 1403 women attending antenatal clinics in the Australian states of New South Wales and Western Australia found that 61% of women consumed alcohol between conception and pregnancy recognition, often at risky levels. Of these women, approximately 30% continued to drink alcohol once they were aware they were pregnant [10]. Similarly, a study of 1570 women attending public antenatal clinics in Melbourne, Australia, found that just over half (54%) of women consumed alcohol in the first trimester, and half of these women continued to consume alcohol throughout the remainder of their pregnancy [8].

Health services providing antenatal care represent an opportune setting to address maternal alcohol consumption during pregnancy. Systematic review evidence shows that psychological and educational interventions for pregnant women may reduce alcohol consumption and increase abstinence from alcohol [11]. Additional evidence suggests that clinician assessment of alcohol consumption and brief interventions, including motivational interviewing, may also reduce the risk of an alcohol-exposed pregnancy [12–18]. Efforts to reduce alcohol consumption during pregnancy in antenatal settings is also acceptable to women, with 97% of Australian women indicating that they wanted information about alcohol use during pregnancy and would be willing to change their alcohol consumption if advised to do so [19]. Consistent with this evidence, international, [20] as well as Australian national [21, 22] and state clinical guidelines [23, 24] recommend that health professionals providing antenatal care use a validated tool to assess alcohol consumption for all pregnant women at the initial visit and throughout the antenatal period, provide brief advice about the potential harms of alcohol consumption during pregnancy and recommend abstinence, and refer women to specialist services if they require assistance to stop consuming alcohol.

Despite clear recommendations in clinical guidelines, implementation of antenatal care addressing maternal alcohol consumption during pregnancy is limited. Internationally, studies show that pregnant women are not routinely assessed for their alcohol consumption during antenatal consultations are not consistently provided with information regarding the effects of alcohol consumption during pregnancy and receive inconsistent advice regarding alcohol intake [25–27]. For instance, in Canada, only 50% of health professionals report providing advice to pregnant women regarding the consumption of alcohol [28] and, in the UK, only two thirds of women reported receiving such advice from a midwife [29]. In Australia, a study of 1143 of health professionals who provide antenatal care found that fewer than half (45%) routinely asked about alcohol consumption during pregnancy, only 25% provided information on the effects of alcohol consumption during pregnancy, and only 13% provided advice consistent with national drinking guidelines [30, 31]. A more recent study involving 166 midwives in Western Australia found that while almost all midwives (93%) asked pregnant women about their alcohol consumption, only 54% used a standardised assessment tool [32].

A small number of studies have been conducted to assess barriers to the provision of care addressing maternal alcohol consumption during pregnancy. These barriers include a lack of systems and/or tools to prompt clinician assessment of alcohol consumption, concerns about patient sensitivity and stigmatisation, lack of staff time, need for staff training, limited access to or knowledge of clinical and patient resources, including culturally appropriate resources for Aboriginal and Torres Strait Islander women, lack of referral options, a perceived lack of skill in delivering care, and a lack of understanding of the importance of providing such care to all women [33–36]. Additionally, the literature on clinical guideline implementation more broadly indicates that other barriers (including commitment to change from organisational leaders/champions, perceived value/need and readiness to change, skills, ability and confidence, and an absence of systems and tools to support/prompt care delivery) commonly impede changes in professional practice [37].

Cochrane reviews of strategies to improve the implementation of recommended clinical practices suggest that a variety of both organisational and individually focused strategies may be effective. These strategies include leadership, local clinical practice guidelines, electronic prompt and reminder systems, local opinion leaders, educational meetings and educational materials, academic detailing, including audit and feedback, and, monitoring the performance of the delivery of healthcare [38–41]. The effectiveness of such strategies in improving the implementation of guideline recommendations is, however, highly variable [38–42] and, to maximise effectiveness, it is recommended that strategies are selected that target specific barriers to the implementation of recommended clinical practices [43–45]. Implementation frameworks such as the Theoretical Domains Framework have been developed to aid the selection of targeted evidence-based implementation strategies [46, 47].

No controlled trials have been conducted to test the effectiveness, cost and cost-effectiveness of implementation strategies in increasing the provision of recommended antenatal care that addresses maternal alcohol consumption during pregnancy. Accordingly, the aim of this study is to examine the effectiveness, cost and cost-effectiveness of a multi-strategy practice change intervention in increasing maternity clinician provision of care addressing the consumption of alcohol by women during their pregnancy.

## Methods

### Study design and setting

**Fig. 1:**
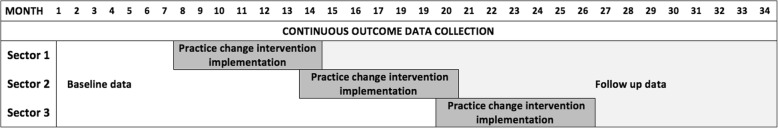
Study design. Figure 1 shows the trial design and implementation of the trial data collection and intervention components over the course of the 34 months trial period. Repeated cross-sectional outcome data from surveys of pregnant women will be gathered on a weekly basis across all three sectors for the duration of the study. Baseline data will be collected for each of the three sectors from 7 months prior to the commencement of the intervention in the first sector to the start of the intervention in each sector. Stepped implementation of a 7-month practice change intervention will be delivered in a randomly selected order at six monthly intervals. Follow-up data will continue to be collected for all three sectors 7 months following completion of the practice change intervention in the third sector

The study will be a randomised stepped-wedge controlled trial design conducted in three sectors (clusters) in the Hunter New England Local Health District in New South Wales, Australia. The sectors are geographically defined groupings of antenatal facilities with common operational management. As shown in Fig. 1, repeated cross-sectional outcome data will be gathered on a weekly basis across all three sectors for the duration of the study (34 months). Baseline (current practice/control phase) data will be collected for each of the three sectors from 7 months prior to the commencement of the intervention in the first sector to the start of the intervention in each sector. Stepped implementation of a 7-month practice change intervention will be delivered in a randomly selected order at six monthly intervals. Follow-up data will continue to be collected for all three sectors 7 months following completion of the practice change intervention in the third sector. The outcomes of the trial will be determined by comparing practice change outcomes between the baseline and follow up periods for the three sectors combined.

A randomised stepped-wedge controlled trial design is recommended for the evaluation of complex practice change interventions in settings such as health services as it provides a number of pragmatic and scientific advantages over a randomised controlled trial design [48, 49]. First, it provides a similar level of evidence as a standard parallel cluster randomised controlled trial (RCT) [50]. Second, although all participants will receive the intervention, its sequential implementation across three sectors provides the capacity to identify secular trends, i.e. changes over time before the intervention is implemented [48]. Third, the design addresses the practical difficulty of recruiting the number of similar antenatal services that would be required for a parallel cluster RCT, instead allowing each cluster to act as its own control [48, 49]. Finally, the design provides an opportunity for all participating services and women to receive the intervention, overcoming ethical and logistical challenges arising from withholding the intervention [50].

Public antenatal services are the largest provider of antenatal health care in Australia, providing services to a diverse population [51]. The antenatal services in the three sectors service urban and rural areas and provide care to over 6000 women annually, accounting for approximately 70% of births in Hunter New England Local Health District public hospitals [52].

### Random allocation and blinding

A statistician who is independent of intervention development and implementation will randomly allocate the order in which the intervention is implemented (stepped) across the three health sectors. The random sequence will be generated using a computerised random number generator with allocation undertaken for all three sectors at the one time. Study personnel involved in collecting outcome data will be blind to the allocated order of the delivery of the intervention across the sectors. Participants providing outcome data will not be informed of the experimental nature of intervention implementation across services and therefore will be blind to the stage of study occurring in the service they attended. Given the practice change nature of the intervention, clinicians in antenatal services will be aware when their service is in the intervention period.

### Participant eligibility and recruitment

#### Antenatal services and clinicians

All public antenatal services in the three sectors will receive the practice change intervention, including midwifery group practices, midwifery clinics, specialist medical services, Aboriginal Maternal Infant Health Services (AMIHS), and multi-disciplinary teams caring for women with complex pregnancies or identified vulnerabilities. The practice change intervention will be provided to all maternity clinicians providing antenatal care in participating services: registered midwives (clinical midwife educators, clinical midwife specialists, clinical midwife consultants, community liaison midwives), medical practitioners (staff specialists in obstetrics, fellows, registrars, resident medical officers, general practice obstetricians), Aboriginal health practitioners, Aboriginal health workers and students. All such clinicians who worked in participating antenatal services for at least one of the 7 months during which the practice change intervention was implemented in their sector will be invited to participate in a post intervention survey.

#### Pregnant women

It is intended that all pregnant women who attend participating services from the start of the practice change intervention in their health sector will receive the intervention. During the 34-month data collection period, women who attend an individual face-to-face antenatal appointment at three time points (i) the time of the first public antenatal service visit (from this point referred to as the ‘booking in’ visit), (ii) 27–28 weeks gestation, or (iii) 35–36 weeks gestation, will be eligible to participate in data collection surveys. To be eligible for participation in such surveys, women need to: be aged 18 years or older, be currently pregnant at more than 12 weeks gestation and less than 37 weeks gestation, have a sufficient level of English language proficiency to complete the survey unaided, and be mentally and physically capable of completing the survey. Women will be ineligible to participate in data collection surveys if they: are determined by clinical discretion to be inappropriate to contact for the survey (e.g. due to medical or social issues); and/or are receiving the majority of their antenatal care via a private obstetrician; and/or have given birth or had a negative pregnancy outcome; and/or had already been selected to participate in the survey for that care time point in the past 4 weeks; and/or had previously declined participation in the survey. The number of women deemed ineligible for the above-listed reasons will be recorded and reported.

Each week, a sample of eligible women who attended an antenatal appointment in the past week (for booking in, 27–28 weeks gestation or 35–36 weeks gestation care) will be randomly selected via a computerised random number generator by members of the research team not involved in delivering care to women. Selected women will be mailed a participant information statement explaining the purpose of the survey 1 week prior to receiving a phone call inviting them to participate in the survey. Study posters will be displayed in antenatal clinics and pamphlets distributed in antenatal information packs provided to all women at the time of their booking in appointment. Women identified via the medical record data as being of Aboriginal or Torres Strait Islander origin and/or women who are attending or enrolled to attend an AMIHS will be first contacted by text message and invited to participate. If they do not respond, they will be followed up with a telephone call 4 days later. All women will have the opportunity to decline participation at any point, including opting out during the clinic visit or when they receive information in the antenatal booking in pack, when they receive the study information letter in the mail, at the time of the phone call or text message, or partway through survey completion. On the morning of the day that a woman is to be contacted via phone call or text message, medical record data will be checked and any women who are identified as having given birth or having had a negative pregnancy outcome will be deemed ineligible and not contacted.

### Intervention

#### Model of care for addressing maternal alcohol consumption in pregnancy

A model of care for addressing alcohol consumption in pregnancy will be implemented in antenatal services across the three participating sectors. The model of care is consistent with international [20] and Australian national [21, 22], and state [23, 24] antenatal clinical practice guidelines and is based on models of assessment and brief intervention that have been shown to reduce the risk of an alcohol-exposed pregnancy [11, 13–18].

**Fig. 2:**
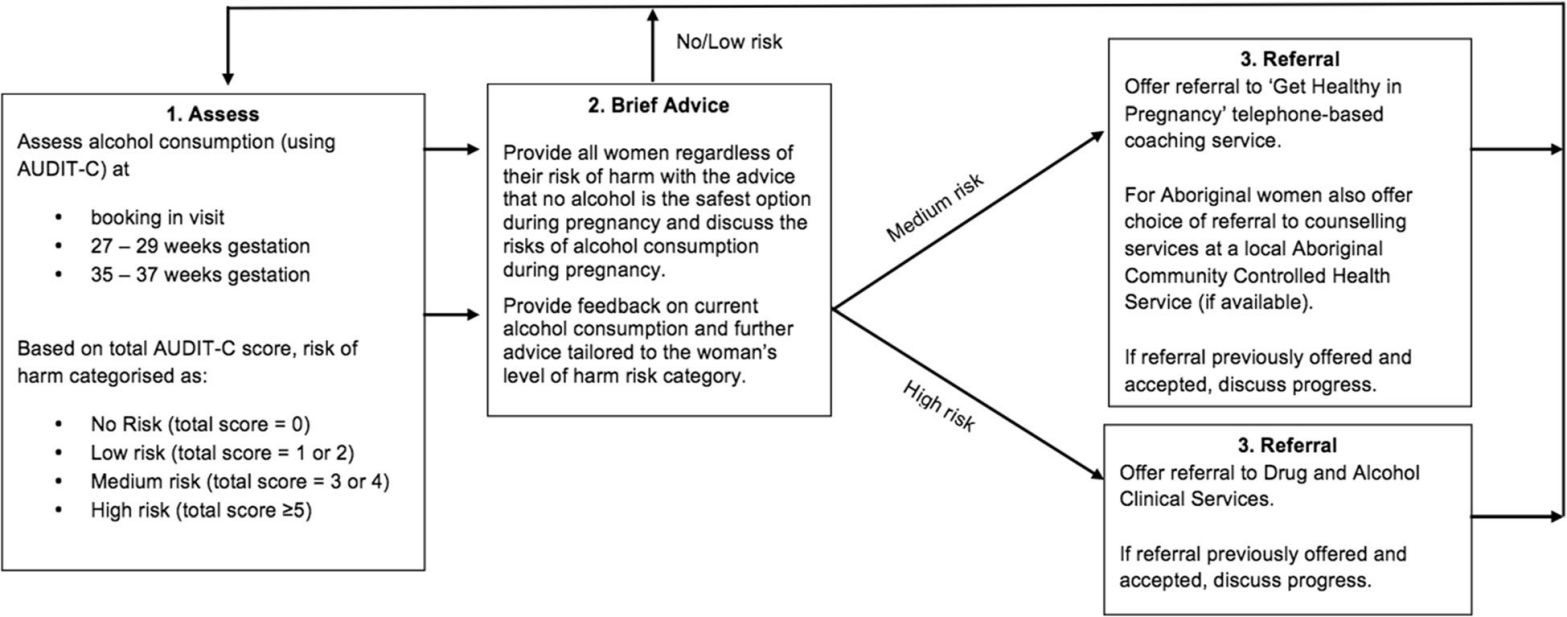
Model of care for addressing maternal alcohol consumption during pregnancy. Figure 2 shows the model of care for addressing maternal alcohol consumption during pregnancy. This model of care will consist of three key elements—assessment, advice and referral—which will be delivered to women who attend an antenatal clinic appointment booking in, 27–29 weeks gestation and 35–37 weeks gestation. The Alcohol Use Disorders Identification Test Consumption (AUDIT-C) tool will be used to assess the alcohol consumption of pregnant women. All women, regardless of their Alcohol Risk of Harm category, will be provided with advice by their maternity clinician that it is best not to consume alcohol at any time during pregnancy and that alcohol consumption during pregnancy can increase risk of harm to the foetus and the woman. Women will be provided with additional advice based on their Alcohol Risk of Harm category. Women with a Medium AUDIT-C risk level will be offered a referral to the Get Healthy in Pregnancy telephone-based coaching service. Aboriginal women with a Medium AUDIT-C risk level will also be offered the option of referral to counselling services at a local Aboriginal Community Controlled Health Service (ACCHS) (if available). For women with a High AUDIT-C risk level, direct referral to Hunter New England Local Health District Drug and Alcohol Clinical Services will be provided. Care from such services will involve ongoing clinical support from a multidisciplinary Drug and Alcohol team throughout pregnancy, including assessment, brief intervention, counselling and withdrawal and post-withdrawal support as clinically indicated

As shown in Fig. 2, the model of care will consist of three key elements–assessment, advice and referral, which will be delivered to women who attend an antenatal clinic appointment the booking in, 27–29 weeks gestation and 35–37 weeks gestation.

1. Assessment of alcohol consumption

The Alcohol Use Disorders Identification Test Consumption (AUDIT-C) tool will be used to assess the alcohol consumption of pregnant women. The tool has shown to be a valid tool for use with pregnant women [53]. Maternity clinicians will ask all women the first AUDIT-C question: ‘How often do you have a drink containing alcohol?’. Those women who provide a response other than ‘Never’, will be asked the remaining two AUDIT-C items: ‘How many standard drinks of alcohol do you have on a typical day when you are drinking?’ and ‘How often do you have five or more alcohol drinks on one occasion?’. These responses will be used to calculate an ‘Alcohol Risk Score’ and ‘Alcohol Risk of Harm’ category (No Risk, Low Risk, Medium Risk, High Risk).

2) Provision of brief advice

All women, regardless of their Alcohol Risk of Harm category, will be provided with advice by their maternity clinician that it is best not to consume alcohol at any time during pregnancy and that alcohol consumption during pregnancy can increase risk of harm to the foetus and the woman. Women will be provided with additional advice based on their Alcohol Risk of Harm category.

3) Referral to specialist services for additional support

Women with a Medium AUDIT-C risk level will be offered a referral to the Get Healthy in Pregnancy telephone-based coaching service. Get Healthy in Pregnancy is a free, state-wide, government-funded telephone coaching service that supports women to make positive health and lifestyle changes [54]. Women can set a goal of alcohol abstinence throughout pregnancy and receive up to ten tailored calls by qualified health coaches. The coaching is based on behaviour change principles designed to assist with goal setting, maintaining motivation and overcoming barriers [54]. Aboriginal women with a Medium AUDIT-C risk level will also be offered the option of referral to counselling services at a local Aboriginal Community Controlled Health Service (ACCHS) (if available). This latter referral pathway was established through consultation with Aboriginal community members and the ACCHS’s that provide care for Aboriginal pregnant women in the study region.

For women with a High AUDIT-C risk level, direct referral to Hunter New England Local Health District Drug and Alcohol Clinical Services will be provided. Care from such services will involve ongoing clinical support from a multidisciplinary Drug and Alcohol team throughout pregnancy, including assessment, brief intervention, counselling and withdrawal and post-withdrawal support as clinically indicated. Referral may also be made to residential drug and alcohol treatment services if required (provided by non-government services).

#### Implementation intervention

A series of organisational and individual clinician focused strategies will be used to support clinician implementation of the model of care.

##### Implementation intervention strategy development

The following staged process was undertaken to develop the implementation strategies:

1. Quantitative anonymous surveys were undertaken with 33 clinicians working in antenatal services across the three sectors and an additional eight managers of maternity services/teams to determine perceived barriers and enablers to their implementation of the model of care for addressing maternal alcohol consumption during pregnancy. The surveys were constructed based on 11 domains of the Theoretical Domains Framework (TDF) [55, 56] and were informed by previous surveys of health professionals [57] and previous studies utilising the TDF across different health care settings [58–60].
2. The priority barriers identified through the clinician and manager surveys were mapped to TDF techniques for behaviour change [43] and a proposed list of implementation strategies was developed based on a review of the literature and advice sought from experts in treatment of alcohol harms, clinical practice change, health service research and behavioural sciences.
3. Consultation with Aboriginal community members, ACCHSs within the participating sectors, AMIHS staff, and Aboriginal population health staff was undertaken to ensure the content of implementation strategies was culturally appropriate for women. Qualitative research (focus groups) was also conducted with Aboriginal mothers (who had attended a public antenatal service in the study region in the last 12 months) and Aboriginal pregnant women to explore experiences of antenatal care in relation to the consumption of alcohol during pregnancy and identify any issues relating to the provision of such care to ensure the implementation strategies were culturally appropriate.
4. Final refinement of implementation strategies and development of strategy content was undertaken following consultation with key clinicians and managers across the three participating health sectors.

##### Implementation intervention strategies

Table 1 lists the implementation strategies that will be used to support the introduction of the model of care for addressing maternal alcohol consumption during pregnancy (Fig. 2), including the evidence source and the TDF domains on which each strategy was based and the barriers that the strategy was intended to address.

**Table 1:** Implementation intervention strategies

Implementation strategy	TDF domain/s [56]	Identified barriers strategy seeks to overcome	Mapped behaviour change techniques [43]	Strategy description
1. Leadership/managerial supervision [65]	• Professional role • Belief about consequences	• Clinician belief that it is not their responsibility to routinely address alcohol consumption during pregnancy. • Clinician and manager belief that there are more important things to do. • Manager belief that they lack support from colleagues to manage staff performance and that staff are resistant to clinical practice change. • Clinician belief that they will not be held accountable if they do not address alcohol consumption. • Clinician belief that their managers do not expect alcohol care to be delivered.	• Social processes of encouragement, pressure and support • Persuasive communication	• Throughout planning and implementation, monthly meetings will be held with management from antenatal services within each of the participating sectors to gather feedback on planned strategies and elicit support. • Antenatal service managers will be asked to distribute key documents/communications to staff and attended all training sessions. • Antenatal service managers will have performance measures related to the model of care added to their operational plans.
2. Local clinical practice guidelines [66]	• Knowledge • Environmental context and resources	• Clinician lack of knowledge of the procedure for addressing alcohol consumption, including referral pathways for women requiring further support. • Clinician feedback that IT systems/forms do not support required care. • Clinician belief that they do not have a clear plan for addressing alcohol consumption during pregnancy and if they have a problem they do not know how to solve it.	• Information regarding behaviour/outcome • Environmental changes • Goal target specified: behaviour or outcome • Contract • Planning and implementation	• A service level guideline and procedure document will detail the required care for addressing alcohol consumption during pregnancy, including assessment, brief advice and referral pathways. • The guidelines and procedure will be uploaded onto the health service’s policy, procedure and guidelines directory and disseminated by service managers to all staff via email and hard copies will be placed in staff common areas.
3. Electronic prompt and reminder system [67]	• Memory, attention and decision processes • Environmental context and resources • Behavioural regulation	• Clinician feedback that they often forget to address alcohol consumption during pregnancy and do not unless the woman expresses it as a priority. • Clinician feedback that IT systems/forms do not support required care. • Clinician feedback that there is a lot to cover in antenatal appointments. • Clinician belief that they do not have a clear plan for addressing alcohol consumption during pregnancy and if they have a problem they do not know how to solve it.	• Environmental changes • Prompts, triggers and cues	• Modifications will be made to existing point-of-care and medical record systems used by maternity clinicians to electronically prompt standardised assessment of alcohol consumption using the validated AUDIT-C alcohol screening tool. Brief advice scripts will be displayed on the point-of-care system based on the woman’s AUDIT-C risk score, as will prompts and tools for referral to appropriate services.
4. Local opinion leaders/ champions [65, 68, 69]	• Social/professional role and identity • Motivation and goals • Social influences	• Clinician belief that it is not their responsibility to routinely address alcohol consumption during pregnancy. • Clinician and manager belief that there are more important things to do. • Clinician belief that other staff do not routinely undertake the model of care and there is no one who can provide support if a problem is encountered. • Manager belief that they lack support from colleagues to manage staff performance and that staff are resistant to clinical practice change.	• Social processes of encouragement, pressure, support • Persuasive communication • Modelling, demonstration of behaviour by others	• Project-specific Clinical Midwife Educators (CMEs) will be appointed to support staff to uptake the model of care and will provide support at a one-on-one, team and service level. The CMEs will be appointed based on their ability to engage and influence staff and model-required behaviours. The role of the CME will be to deliver and monitor each of the implementation support strategies and be responsive to the specific implementation needs of each antenatal service. • Additional local antenatal clinical leaders will be engaged to provide encouragement and demonstration of required behaviours in each antenatal services as required (e.g. for specific professional disciplines).
5. Educational meetings and educational materials [70, 71]	• Knowledge • Skills • Beliefs about capabilities • Beliefs about consequence • Environmental context and resources • Emotion	• Clinician lack of knowledge in the procedure for addressing alcohol consumption, including referral pathways for women requiring further support. • Clinician lack of skill in assessing alcohol consumption during pregnancy using a validated tool and offering referrals to women requiring further support. • Clinician lack of training in addressing alcohol consumption according to guidelines. • Clinician belief that they have limited capability to assess alcohol consumption during pregnancy using a validated tool and offer appropriate referrals. • Manager belief that they have limited capability to competently use performance monitoring tools and reports and have conversations with staff regarding performance. • Clinician belief that pregnant women will react negatively if asked about alcohol consumption and that it will have a negative impact on their client-clinician relationship. • Clinician feedback that they are hesitant to address alcohol due to child protection implications. • Clinician belief that they do not have access to appropriate information resources and there is a lack of support services to refer women to. • Clinician lack of confidence in addressing alcohol consumption in appointment time, when other clinicians are present and when women show lack of interest. • Clinician feedback that they feel nervous and uncomfortable addressing alcohol consumption with women.	• Goal/target specified behaviour or outcome • Increasing skills- through problem solving, decision-making, goal-setting • Rehearsal of relevant skills • Modelling/ demonstration of behaviour by others • Perform behaviour in different settings • Social process of encouragement/ pressure support • Persuasive communication • Information regarding behaviour/ outcome • Coping skills	• Training will be provided to all antenatal service clinicians via a 30-minute online training module and face-to-face sessions. Content will be adapted from the accredited ‘Women Want to Know’ courses [72]. The CME will facilitate clinicians completing the online training and coordinate face-to-face training sessions, which will be rostered into routine clinical meetings and include, lecture style sessions, interactive, case-study based sessions and one-on-one sessions. • Training content will include: − The effects of alcohol consumption during pregnancy and associated health outcomes. − Guideline recommendations for alcohol consumption during pregnancy. − Prevalence of alcohol consumption by pregnant women. − The model of care for addressing alcohol consumption during pregnancy: 1) alcohol consumption assessment; 2) brief advice; and 3) referral for ongoing care. − Effectiveness and acceptability of addressing alcohol consumption during pregnancy in routine antenatal care. − Culturally appropriate practices when addressing alcohol consumption with Aboriginal women. • Clinicians will also be provided with written resources (hardcopy and electronic) to support the model of care, including standard drink measure charts and point-of-care written prompts/reminders (e.g. stickers in charts).
6. Academic detailing, including audit and feedback [45, 73, 74]	• Behavioural Regulation • Skills • Beliefs about consequences • Social Influences	• Clinician belief that they do not have a clear plan for addressing alcohol consumption during pregnancy and if they have a problem they do not know how to solve it. • Clinician lack of skill in addressing alcohol consumption according to guidelines. • Manager belief that additional burden will be placed on clinicians, that staff will react negatively if performance is discussed and that staff will not take on board feedback about performance. • Manager feedback that it is difficult to release clinicians from clinical work to attend training. • Clinician belief that pregnant women do not expect alcohol to be addressed in antenatal appointments.	• Goal/target specified behaviour or outcome • Monitoring • Contract • Planning, implementation • Increasing skills- problem solving, decision-making, goal-setting • Rehearsal of relevant skills • Social process of encouragement/pressure support • Feedback • Persuasive communication • Information regarding behaviour and outcome	• Data from both medical records and telephone surveys conducted with women who attended the antenatal services will be used to provide feedback on levels of care provision for addressing alcohol consumption during pregnancy. • Data will routinely be fed back to antenatal service teams by the CME. The CME will visit service teams in their antenatal clinics to support discussion of the feedback and development of action plans in response to such in order to improve care. • Women’s acceptability of their antenatal service team providing each of the care elements will also be fed back.
7. Monitoring and accountability for the performance of the delivery of healthcare [73]	• Social Influences • Beliefs about capabilities • Environmental context and resources • Memory, attention, decision processes	• Clinician belief that their managers do not expect alcohol care to be delivered. • Manager belief that they have limited capability to competently use performance monitoring tools and reports and have conversations with staff regarding performance. • Manager feedback that they do not have adequate data entered from staff to use for performance measurement, have competing work tasks and do not have the supports/resources to manage performance. • Managers’ feedback that they forget about tools to manage performance and are less likely to manage performance of staff resistant to change. • Manager feedback that it is stressful to manage performance.	• Social processes of encouragement, pressure, support • Environmental changes • Contract • Prompts, triggers, cues • Feedback	• Antenatal service managers will be supported by the CME to report, interpret and monitor performance measures for the model of care for addressing alcohol consumption during pregnancy. The CME will also support these mangers to disseminate these results to their antenatal service staff through team meetings, emails and other usual communication mechanisms. • Performance measures will be built into the existing monitoring and accountability frameworks for antenatal services, including service-level operational plans and performance measures at service manager and team manager level.

##### Implementation intervention delivery timeline

The intervention strategies listed above will be implemented in each sector for 7 months prior to follow up data collection (Fig. 1). This will include a 1-month period introducing the practice change and a 6-month intensive practice change intervention. Given their organisational and system focus, all strategies, other than the local opinion leader (Clinical Midwife Educator (CME)) and academic detailing strategies, have the potential to continue to be implemented following the 7-month study intervention period, subject to the operational decisions of the Local Health District.

### Control and contamination

#### Usual care

Prior to implementation of the practice change intervention in each of the three sectors, usual antenatal care for addressing maternal alcohol consumption during pregnancy will be provided. Such care is likely to vary by antenatal service and clinician as no existing health sector-wide guideline or procedure specifies the provision of routine care for addressing alcohol consumption during pregnancy.

#### Potential for contamination

As the research team will control the initiation and delivery of all the intervention elements, the intervention strategies will not be accessible to antenatal clinicians during the baseline (control) phase. Although potential for contamination during this phase from staff movement between sectors is possible, it is considered to be limited due to the structural and systemic nature of the implementation strategies. Information on movement of clinicians between participating sectors will be collected throughout the study.

### Measures

#### Primary trial outcomes

There are four primary outcomes for this trial. They are the proportion of all antenatal clinic appointments (at ‘booking in’, 27-28 weeks gestation and 35–36 weeks gestation) for which women report:

1. Being assessed for alcohol consumption and level of risk using the AUDIT-C.
2. Being provided with brief advice related to alcohol consumption during pregnancy.
3. Receiving, relative to their level of risk, the relevant elements of antenatal care for addressing alcohol consumption during pregnancy (advise and refer).
4. Being assessed for alcohol consumption and level of risk using the AUDIT-C and receiving, relative to their level of risk, the relevant elements of antenatal care for addressing alcohol consumption during pregnancy (advise and refer).

#### Secondary trial outcome

For women attending antenatal appointments at ‘booking in’, 27–28 weeks gestation and 35–36 weeks gestation, alcohol consumption since pregnancy recognition as measured by total AUDIT-C score will be collected based on self-report of women. AUDIT-C is a validated tool for assessing risk of harm due to alcohol consumption [53].

#### Process evaluation measures

The acceptability, appropriateness, feasibility, intervention fidelity and reach of the model of care for addressing maternal alcohol consumption in pregnancy and the implementation strategies will be assessed via surveys of women and clinicians and project records. These process measures will be based on an implementation evaluation framework [61] and use validated measures where available [62]. Measures to assess implementation intervention reach will include participation of antenatal clinical staff in educational meetings, interaction with local opinion leaders, involvement in academic detailing/ audit and feedback sessions, and receipts of clinical practice guidelines. To determine reach of the implementation intervention strategies across different groups of clinicians, data will be collected from clinicians on position/profession, level of training, and length of time working in current antenatal service and in antenatal services generally. To assess delivery of the model of care across different demographic groups of women, the following information will be collected from pregnant women: age, gender, highest level of education, employment status, geographical location, Aboriginal or Torres Strait Islander status of woman and baby, household composition, current gestation, gestation at first antenatal clinic visit, whether attending care for first or subsequent pregnancy and alcohol consumption prior pregnancy (via AUDIT-C).

#### Cost and cost-effectiveness

To provide a measure of the investment required to develop, implement and maintain the effect of the implementation strategies, resource use will be prospectively measured and valued from a public finance perspective. The outcomes from the cost analysis will be (i) an estimate of the cost required to develop the intervention strategies, (ii) the net cost of delivering the implementation intervention (including labour costs for the CME and the clinicians to conduct/participate in each of the implementation strategies, undertaking quality assurance processes, providing managerial oversight), and (iii) assuming a positive trial outcome, the expected incremental cost to maintain effect. Additionally, the cost-effectiveness of the implementation strategies will be assessed relative to the baseline (current practice) phase in each of the three sectors. The incremental cost-effectiveness ratio (ICER) will be calculated as the difference in average cost between the intervention and baseline phases, divided by the difference in the primary outcome. Sensitivity analyses will test the robustness of results to selected issues and assumptions.

### Data collection procedures

#### Primary and secondary outcome measures

Each week a sample of women who in the past week attended an antenatal clinic for a ‘booking in’ visit, a visit when they were 27–28 weeks gestation, or a visit when they were 35–36 weeks gestation will be sent a letter providing information about the study and inviting them to participate in a computer assisted telephone interview (CATI) survey. Telephone contact will be attempted with women up to ten times over a 2-week period, including at different times of the day and on weekdays and weekends, in order to elicit consent and completion of the survey. If a woman declines to participate in the CATI, they will be invited to complete the survey online. If they consent to participate in the online survey, they will be sent a survey link via text message. Women who are of Aboriginal or Torres Strait Islander origin and/or are attending or enrolled to attend an AMIHS will be offered via text message the choice of participating in the survey via either CATI or online mode.

The CATI survey will be conducted by experienced female interviewers who will receive specific training and undertake practice interviews. The CATI and online survey script are identical in the wording of questions, response options and help provided. Both surveys will be pilot tested prior to starting the study to test comprehension, logic flow and survey length.

#### Process evaluation

Data for the aforementioned process evaluation measures will be collected via surveys with women (as per procedure described above) and clinicians. Online surveys of clinicians will be conducted at the completion of the intervention in each sector. All eligible clinicians in antenatal services in the participating sectors will be sent a link to an online survey via email, and also given the option to complete the survey on tablet computers/laptops in regular in-services and clinic meetings. Surveys will be completed anonymously. Additional process data to assess intervention fidelity and reach will be collected using project management logs completed by project staff.

#### Cost and cost effectiveness

Project management logs, including a cost capture template, will be used to prospectively collect data regarding the resources expended during intervention development and implementation.

#### Overall data management

Management of trial data will be in accordance with a data management protocol, which has been developed and approved by the project’s advisory group. Data will be stored securely as per the requirements of the Hunter New England Human Research Ethics Committee, The University of Newcastle Human Research Ethics Committee and the Aboriginal Health and Medical Research Council. Data will only be accessible to primary researchers and statisticians. Confidential participant data will be stored securely and not linked to survey responses.

### Sample size and power calculations

It is expected that 70% of invited women will consent to participate in the surveys (based on previous work by the research team [63]). Assuming 48 working weeks a year, and an intra-class correlation of 0.01, a sample of 200 women per month (approximately 67 women per each of the three time points: booking in visit, 27–28 weeks gestation, 35–36 weeks gestation) will give the study 80% power to detect an absolute increase in care provision of 15% in the intervention period (based on a conservative 50% estimate of prevalence of care provision at baseline) in at least one of the four primary outcomes at a 1.25% significance threshold. Assuming there are approximately 190 women eligible per week, a weekly sample of 72 women (i.e. 24 women per time point) with a 70% survey completion rate (*n* = 50) will result in the required number of women needed per month.

### Statistical analysis

Baseline and follow-up primary outcomes data will be analysed using a logistic mixed model to detect change over time in the reported receipt of recommended antenatal care for addressing alcohol consumption during pregnancy. For the secondary trial outcome, linear mixed models will be used to analyse changes in AUDIT-C scores of participants between baselines and follow up periods. The models will have a period term (fixed effect, reflecting pre-post difference, the main indicator of effect) and health sector term (fixed effect). Where appropriate, the models will also include fixed effects for client group (booking in, 27–28 weeks gestation, 35–36 weeks gestation) and a time term (fixed effect, to pick up any secular trend). In the latter models, a client group by period term will detect differences between the client groups in their response to the intervention. Where the interaction terms are significant, subgroup analyses will be reported for each of the three client groups. Descriptive statistics will be used to report on process measures and interventions costs. Process outcomes will be used to narratively interpret the results of primary outcome analysis. SAS (V9.3 or later) will be used for all statistical analyses.

### Research trial governance

A research co-production approach has been employed in the development and design of the study [64]. The conduct of the study will similarly be overseen by an advisory group consisting of researchers, policy makers, practitioners and clinical experts with expertise related to alcohol, health promotion, implementation science, FASD, obstetrics and maternal health. A project team consisting of research staff and practitioners will develop and operationalise implementation strategies and data collection components of the trial according to study protocol. Local clinical experts based at each of the three participating sectors will provide advice on aspects of the model of care and implementation strategies that require sector-specific tailoring.

### Aboriginal cultural governance

A series of Aboriginal cultural governance task groups, co-led by Aboriginal and non-Aboriginal staff, will provide guidance on cultural considerations for Aboriginal and Torres Strait Islander people relating to the model of care, implementation strategies, data collection, and interpretation and dissemination of study findings.

### Trial discontinuation or modification

There are no criteria for trial discontinuation as it is not anticipated that any events would occur that would warrant discontinuing the trial. Any unforeseen adverse events will be reported to the Hunter New England Human Research Ethics Committee (primary approval committee) and advice sought regarding required action. The trial registration record will be updated with any protocol modifications and any deviations from original protocol will be reported in study outcome papers.

## Discussion

Despite the need, there is a clear absence of research evidence of the effectiveness, cost and cost-effectiveness of implementation strategies to improve antenatal care that addresses maternal alcohol consumption during pregnancy. This will be the first randomised controlled trial to evaluate the effectiveness of such intervention strategies. The stepped-wedge design is feasible and acceptable in the context of conducting a trial across multiple antenatal services. The study has strong design elements including random allocation of the order of strategy implementation across the health sectors and blinding of data collection staff. The implementation intervention strategies have been developed based on key implementation science frameworks and using data from surveys with antenatal services staff and managers. A research co-production approach has been employed in the design of the study and will be employed in its conduct and dissemination.

If positive changes in clinical practice are found, the study will provide evidence to support the delivery by health services of the implementation strategies to improve antenatal care addressing this recognised risk to the health and wellbeing of both the mother and child. The methods used in this trial have the potential to provide a framework for the development of initiatives for improving the implementation of models of care, both in the antenatal clinic setting and in other clinical environments.

## Abbreviations

ACCHS: Aboriginal Community Controlled Health Service
AMIHS: Aboriginal Maternal Infant Health Service
AUDIT-C: Alcohol Use Disorders Identification Test-Consumption
CATI: Computer-assisted telephone interview
CME: Clinical Midwife Educator
FASD: Fetal alcohol spectrum disorder
ICER: Incremental cost-effectiveness ratio
RCT: Randomised controlled trial
TDF: Theoretical Domains Framework
